# Proteomic Analysis of Primary Graft Dysfunction in Porcine Lung Transplantation Reveals Alveolar-Capillary Barrier Changes Underlying the High Particle Flow Rate in Exhaled Breath

**DOI:** 10.3389/ti.2024.12298

**Published:** 2024-04-08

**Authors:** Anna Niroomand, Gabriel Hirdman, Nicholas Bèchet, Haider Ghaidan, Martin Stenlo, Sven Kjellström, Marc Isaksson, Ellen Broberg, Leif Pierre, Snejana Hyllén, Franziska Olm, Sandra Lindstedt

**Affiliations:** ^1^ Wallenberg Centre for Molecular Medicine, Faculty of Medicine, Lund University, Lund, Sweden; ^2^ Department of Clinical Sciences, Faculty of Medicine, Lund University, Lund, Sweden; ^3^ Lund Stem Cell Center, Faculty of Medicine, Lund University, Lund, Sweden; ^4^ Rutgers Robert Wood Johnson University Hospital, New Brunswick, NJ, United States; ^5^ Department of Cardiothoracic Surgery and Transpantation, Skåne University Hospital, Lund, Sweden; ^6^ Department of Cardiothoracic Anaesthesia and Intensive Care, Skåne University Hospital, Lund, Sweden; ^7^ Department of Clinical Sciences, BioMS, Lund, Sweden

**Keywords:** primary graft dysfunction, lung transplantation, particle flow rate, exhaled breath particles, mass spectrometry

## Abstract

Primary graft dysfunction (PGD) remains a challenge for lung transplantation (LTx) recipients as a leading cause of poor early outcomes. New methods are needed for more detailed monitoring and understanding of the pathophysiology of PGD. The measurement of particle flow rate (PFR) in exhaled breath is a novel tool to monitor and understand the disease at the proteomic level. In total, 22 recipient pigs underwent orthotopic left LTx and were evaluated for PGD on postoperative day 3. Exhaled breath particles (EBPs) were evaluated by mass spectrometry and the proteome was compared to tissue biopsies and bronchoalveolar lavage fluid (BALF). Findings were confirmed in EBPs from 11 human transplant recipients. Recipients with PGD had significantly higher PFR [686.4 (449.7–8,824.0) particles per minute (ppm)] compared to recipients without PGD [116.6 (79.7–307.4) ppm, *p* = 0.0005]. Porcine and human EBP proteins recapitulated proteins found in the BAL, demonstrating its utility instead of more invasive techniques. Furthermore, adherens and tight junction proteins were underexpressed in PGD tissue. Histological and proteomic analysis found significant changes to the alveolar-capillary barrier explaining the high PFR in PGD. Exhaled breath measurement is proposed as a rapid and non-invasive bedside measurement of PGD.

## Graphical Abstract

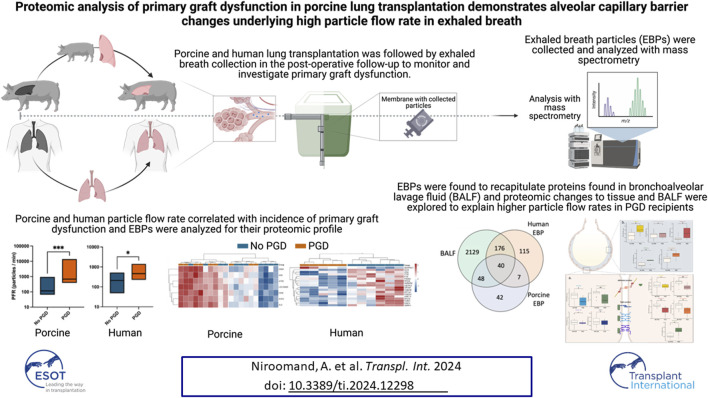

## Introduction

Primary graft dysfunction (PGD) remains a challenge in the postoperative management of lung transplantation (LTx) with an estimated incidence rate of up to 25% of all cases [1]. Recognition and appropriate management are particularly important as PGD grade 3 correlates with increased mortality and rates of chronic lung allograft dysfunction (CLAD) [2–4].

While PGD is readily diagnosed by the PaO_2_/FiO_2_ ratio and chest imaging, current diagnostic tools do not necessarily indicate early onset, offer a means of non-invasive bedside detection, or provide a more detailed view of disease pathology. Chest x-rays, while non-invasive and readily available, are not necessarily specific and do not preclude the existence of other processes. Advanced techniques for patient evaluation such as bronchoalveolar lavage and transbronchial biopsy are invasive and have associated risks. Sampling of exhaled breath particles (EBPs), in contrast, utilizes a non-invasive device connected to the mechanical ventilation circuit with no additional safety considerations. The benefit of this form of sampling would be the ability to quickly identify PGD while being able to analyze the patient’s condition from a more granular perspective given the downstream analyses available for EBP collection.

The efficacy of EBP collection as a methodology has previously been demonstrated in patients in intensive care units (ICUs) and post-transplant patients on mechanical ventilation to show feasibility and lack of adverse effects [5–8]. Porcine models have also demonstrated a relationship between lung injury and particle flow rate [9]. EBPs are thought to originate from the distal respiratory tract lining fluid (RTLF) as the small airways open and close [10–12] and share a similar composition to bronchoalveolar lavage fluid or BALF [10, 11, 13]. While the safety of EBP collection has been proven, its proteomic composition and the mechanism by which disease processes lead to higher PFR have not yet been elucidated, motivating the current study. Additionally, there are few proteomic studies of lung transplantation in general and PGD in particular, with the majority focusing on biomarkers in the blood or BALF [14–17]. Even without considering PGD, studies of the proteome in lung tissue specifically following transplantation are severely limited, with a literature search revealing only one other study examining proteins in post-transplant porcine tissue [18]. Consequently, there is a great need to gain a more detailed understanding of PGD using a variety of profiles sourced from tissue, BALF, and exhaled breath, all of which would be valuable in understanding disease pathogenesis. The comparison of EBP proteins with BALF and tissue proteomes would also validate the collection of exhaled breath as a clinically valuable monitoring tool.

In this study, we utilized a pig lung transplantation model as a platform to study PGD. We applied particle flow rate (PFR) measurement and EBP collection to postoperative mechanical ventilation and correlated PFR with disease occurrence. We then isolated and identified the proteins found in the EBP, validating the methodology for the detection of PGD and compared BALF and tissue proteins to porcine and human lung transplant EBPs. We utilized the proteomic findings to understand the mechanism of higher PFR in PGD. We hypothesized that the epithelial and endothelial damage that occurs in PGD underlies the particle accumulation in the RTLF behind the higher PFR in the disease state.

## Materials and Methods

Further details are provided in the online Supplementary Materials and Methods.

### Ethical Considerations for Porcine Experiments

The study was approved by the local Animal Research Ethics Committee (Dnr 5.2.18-4903/16, and Dnr 5.2.18-8927/16) at Lund University. All animals received care according to the USA Principles of Laboratory Animal Care of the National Society for Medical Research, Guide for the Care and Use of Laboratory Animals, National Academies Press (1996). All human patients signed written informed consent and approval was obtained from the Ethics Committee for Research (Dnr 2017/396).

### Animal Preparation

**FIGURE 1:**
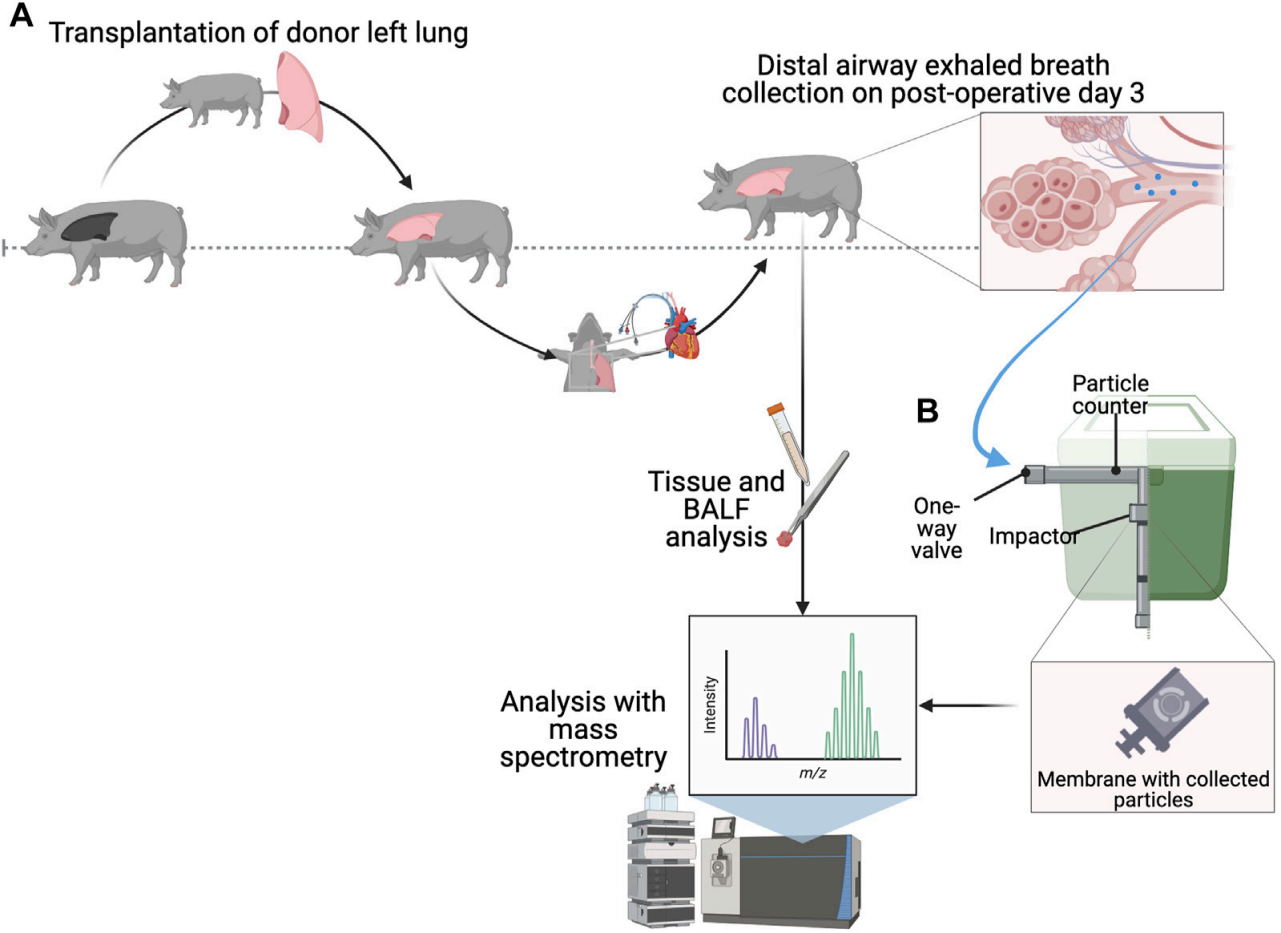
Experimental overview and observed particle flow rate (PFR) in recipients with primary graft dysfunction (PGD). **(A)** Overview of transplantation followed by the right pneumonectomy and distal airway exhaled breath collection on postoperative day 3. **(B)** Schematic illustrating the collection of particles from the distal airways, where exhaled breath travels through a one-way valve in the particles in exhaled air (PExA) device, which counts them prior to impaction. Samples including tissue, bronchoalveolar lavage fluid (BALF) and collected exhaled breath particles were then analyzed for protein identity using mass spectrometry. Figure created in biorender.com.

An overview is provided in Figures 1A, B. All donors (*n* = 22) and recipients (*n* = 22) were premedicated with xylazine (Rompun^®^ vet. 20 mg/mL; Bayer AG, Leverkusen, Germany; 2 mg/kg) and ketamine (Ketaminol^®^ vet. 100 mg/mL; Farmaceutici Gellini S.p.A., Aprilia, Italy; 20 mg/kg). All animals were placed under general anesthesia with ketamine (Ketaminol^®^ vet, 100 mg/mL; Farmaceutici Gellini S.p.A., Aprilia, Italy; 20 mg/kg), midazolam (Midazolam Panpharma^®^, Oslo, Norway) and fentanyl (Leptanal^®^, Lilly, France). A pulmonary artery catheter (Swan-Ganz CCOmbo V and Introflex, Edwards Lifesciences Services GmbH, Unterschleissheim, Germany) was inserted into the right internal jugular vein and an arterial line (Secalon-T™, Merit Medical Ireland Ltd., Galway, Ireland) was placed in the right common carotid artery.

### Lung Transplantation and Monitoring

Lung harvesting from the donor and transplantation into the recipient followed the previous descriptions [19]. Recipient care followed clinical standards and immunosuppression, infection prophylaxis and ventilatory strategies are described in the Supplementary Methods. On day 3 post-transplantation, a right pneumonectomy (including the accessory lobe) allowed for assessment of the transplanted left lung (Figure 1A). The recipient was followed for an additional 4 h under one-lung ventilation, with tidal volume and respiratory rate adjusted to maintain a peak pressure <30 cmH_2_O. All recipients were monitored throughout the post-transplantation period with hemodynamic parameters and arterial blood gases (ABL 90 FLEX blood gas analyzer, Radiometer Medical ApS, Brønshøj, Denmark) analyzed hourly.

### Particles in Exhaled Air (PExA) and Exhaled Breath Particles (EBP)

Following the right pneumonectomy, a customized PExA 2.0 device (PExA, Gothenburg, Sweden) was connected to the expiratory limb of the ventilator, as previously described [6, 9] to measure PFR (particles per minute or ppm) and deposit particles on a membrane (Figure 1B). Membranes were kept frozen at −80°C until analysis.

### Staging of Primary Graft Dysfunction

PGD was staged on postoperative day 3 based on the PaO_2_/FiO_2_ ratio according to the guidelines of the International Society for Heart and Lung Transplantation (ISHLT) [20]. Chest imaging was performed with a mobile C-arm x-ray machine (Siemens, Munich, Germany).

### Histopathological and Immunofluorescence Analyses

Baseline biopsies were taken from the right lower lobe after intubation and from the transplanted left lung at the completion of the experiment. All biopsies were fixed in a 10% neutral buffered formalin solution (Sigma-Aldrich, Merck KGaA, Darmstadt, Germany). For histopathological analysis, sections were stained with hematoxylin and eosin (Merck Millipore, Darmstadt, Germany). Images from each recipient were assessed by two blinded scorers to report a lung injury score. For immunofluorescence imaging, sections stained with 4′,6-diamidino-2-phenylindole (DAPI), Lycopersicon Esculentum lectin (LEA) DyLight-488 and aquaporin-5 (AQP-5) were imaged on a Nikon A1RHD confocal microscopy platform (Nikon, Tokyo, Japan). Alveoli were individually imaged at random locations and analyzed using Fiji software [21]. A morphological quotient or MQ was calculated by dividing the alveolar circularity by its wall thickness.

### Collection of EBPs From Human Lung Transplantation Patients

Membranes with EBPs were collected from 11 lung transplant recipients in the ICU using a modified PExA 2.0 instrument, as previously described [5]. PGD was graded according to the ISHLT guidelines based on arterial blood gas measurements, ventilator settings, and imaging. All patients arrived at the ICU after transplantation with a 7.5-mm tracheal tube and were ventilated according to unit guidelines, including a tidal volume of 6 mL/kg, positive end-expiratory pressure of 5 cmH_2_O, end-inspiratory pressure of less than 25 cmH_2_O, and an inspiratory to expiratory ratio of 1:2 (Maquet Servo I, Getinge, Solna, Sweden). EBPs were collected during measurements taken over 2 h from the second or third post-operative day based on the last measurement possible while the patient was on mechanical ventilation.

### Mass Spectrometry Analysis

Proteins were extracted from porcine tissue, porcine BALF, porcine EBP membranes, and human EBP membranes. Mass spectra were acquired using a data-independent acquisition (DIA) method and analyzed with DIA-NN v 1.8.1 [22]. After quality control, two porcine EBP membranes, and two porcine BALF samples were excluded from further analysis. Differentially expressed proteins were determined with a threshold *p*-value <0.05 using the log2-transformed label-free quantification (LFQ) intensities and fold-change thresholds estimated from bootstrapping procedures. *p*-values were adjusted to determine an FDR-adjusted q-value with a significance level of 0.05. Hierarchical clustering in the heat map was performed on normalized, log2-transformed LFQ intensities (z-scores). The log2-fold change was used as the differential rank statistic for the gene set enrichment analysis (GSEA). A protein analysis through evolutionary relationships (PANTHER) overrepresentation test^1^ of all EBP proteins was performed to look for statistically significantly enriched gene ontology (GO) terms under the biological process ontology.

### Calculations and Statistics

Continuous variables were reported as median and interquartile range (IQR). Statistically significant differences were tested with the Student’s t-test and ANOVA for normally distributed data and with the Mann-Whitney test and the Kruskal-Wallis tests for non-normally distributed data. A Chi-squared test was performed to analyze the observed frequencies of categorical variables. All statistical analyses were performed using GraphPad Prism 9.1 and R Studio (version 4.2.2). Significance was defined as *p* < 0.001 (***), *p* < 0.01 (**), *p* < 0.05 (*), and *p* > 0.05 (not significant).

## Results

### Human Patient Demographics and Characteristics

Within the cohort, four patients were transplanted for idiopathic pulmonary fibrosis, four for chronic obstructive pulmonary disease, and another three for cystic fibrosis; none had an active smoking status. Following transplantation, 6 patients had PGD grade 0 while 5 patients had PGD grade 2. In the non-PGD group, the median age was 61 years (56–64.5) and all were men. The non-PGD group had a median pH of 7.44 (7.40–7.46), lactate of 2.1 (1.85–2.9), and ventilation with a median tidal volume of 522 mL (497–571), a median minute ventilation of 9.8 (8.9–10.8), and a median PEEP of 5 cmH_2_O (5–5). In the PGD group, the median age was 56 years (55–59) and 3 of the 5 patients were women. The PGD group had a median pH of 7.40 (7.37–7.44), lactate of 1.6 (1.4–2.3), and was ventilated with a median tidal volume of 454 mL (444–548), median minute ventilation of 9.0 (8.1–10.8), and a median PEEP of 5 cmH_2_O (5-5).

### Primary Graft Dysfunction After Porcine Lung Transplantation Correlates With Histologic Analysis

**FIGURE 2:**
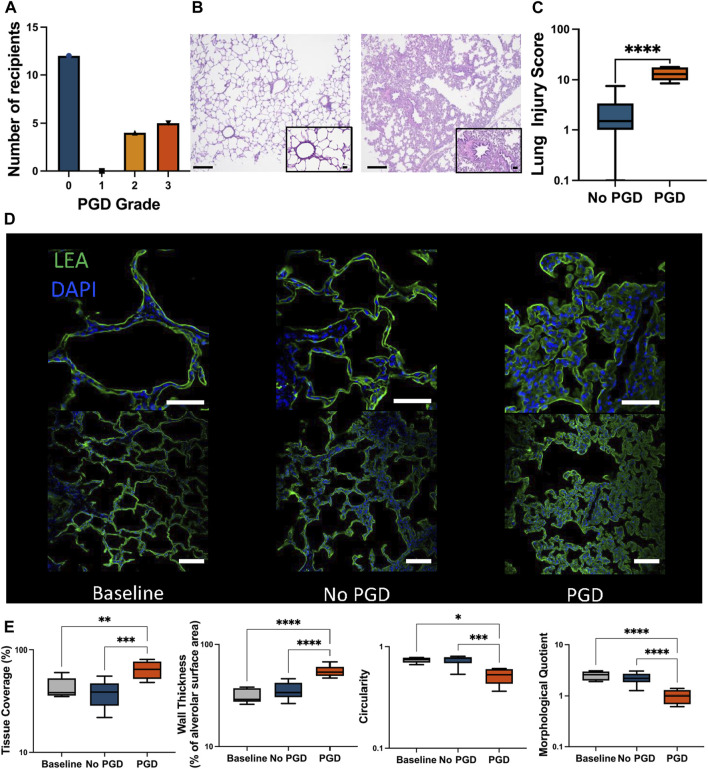
Incidence of primary graft dysfunction (PGD) in lung transplant recipients. **(A)** PGD grade was determined according to the ISHLT guidelines and recipients were graded 0–3 based on the PaO_2_/FiO_2_ ratio and chest imaging. **(B)** Representative images of hematoxylin and eosin (H&E) staining of biopsies taken at the experimental endpoint show the differences between tissue from recipients without PGD (left) and those with PGD grades 2-3 (right). The scale bar in the larger image represents 0.5 mm and the callout shows a magnified portion of tissue where the bar represents 0.2 mm. **(C)** Blinded scoring of the H&E biopsies taken at the experimental endpoint. **(D)** Representative images of immunofluorescence staining for Lycopersicon esculentum lectin (LEA, green) and 4′,6-diamidino-2-phenylindole (DAPI, blue) at the lung biopsies taken at baseline prior to transplantation (left), at the experimental endpoint from a recipient without PGD (center) and at the experimental endpoint from a recipient with PGD (right). The top row shows a magnified portion of the tissue in the bottom row. The scale bar in the top row represents 50 μm while the bar in the bottom row represents 100 μm. **(E)** Morphological analysis of the immunofluorescence staining was conducted on the biopsies taken at baseline prior to transplantation, and the experimental endpoints for PGD and no PGD recipients post-transplantation on LEA and DAPI staining, showing the percent of tissue coverage in each image field of view (far left), in addition to the average thickness of individual alveoli (second from left), the calculated alveolar circularity (third from left), and the morphological quotient calculated based on the wall thickness and circularity (right). Plots represent samples taken from baseline biopsies (*n* = 5), the experimental endpoint for recipients with PGD (*n* = 9) or without PGD (*n* = 13). Values represent the median and interquartile range (box) with minimum and maximum values (whiskers). For statistical comparisons between two groups the two-tailed Mann-Whitney test was used. For comparisons between more than two groups a one-way ANOVA or Kruskal-Wallis test was used, followed by Tukey’s or Dunn’s *post hoc* tests, respectively. *p < 0.05, **p < 0.01, ***p < 0.001, ****p < 0.0001.

All porcine recipients underwent a left LTx and were monitored for 3 days, after which a right pneumonectomy was performed to monitor the isolated left transplanted lung. Porcine recipients were assessed for PGD according to ISHLT guidelines. Severe PGD with grades 2 and 3 was detected in nine recipients while the remaining twelve had PGD grade 0 (Figure 2A).

PGD grades were correlated with the histological examination of end-experiment lung biopsies (Figure 2B). PGD samples received a lung injury score of 13.00 (9.75–17.75) compared to 1.50 (1.00–3.38) in non-PGD samples (*p* < 0.0001, Figure 2C). Signs of damage including immune cell infiltration, alveolar wall thickening, and capillary congestion were noted.

Immunofluorescence morphological examination showed increased damage in PGD (Figure 2D). Tissue coverage was significantly higher in the PGD group compared to the non-PGD group and the pre-transplant baseline (*p* < 0.0001 and *p* < 0.0001 respectively, Figure 2E). Alveolar walls were significantly thicker and alveolar circularity was significantly decreased (*p* < 0.0001, *p* = 0.0009, Figure 2E). The morphologic quotient (MQ) which takes into account the contribution of structural changes in the alveolar wall and circularity showed significant damage compared to the baseline and the non-PGD groups (*p* < 0.0001 and *p* < 0.0001 respectively, Figure 2E). The MQ of the non-PGD group did not significantly differ from the baseline (*p* = 0.506).

### PGD Incidence Correlates With Higher Rates of Particle Flow and Particles Revealed a Proteomic Profile Similar to BALF

**FIGURE 3:**
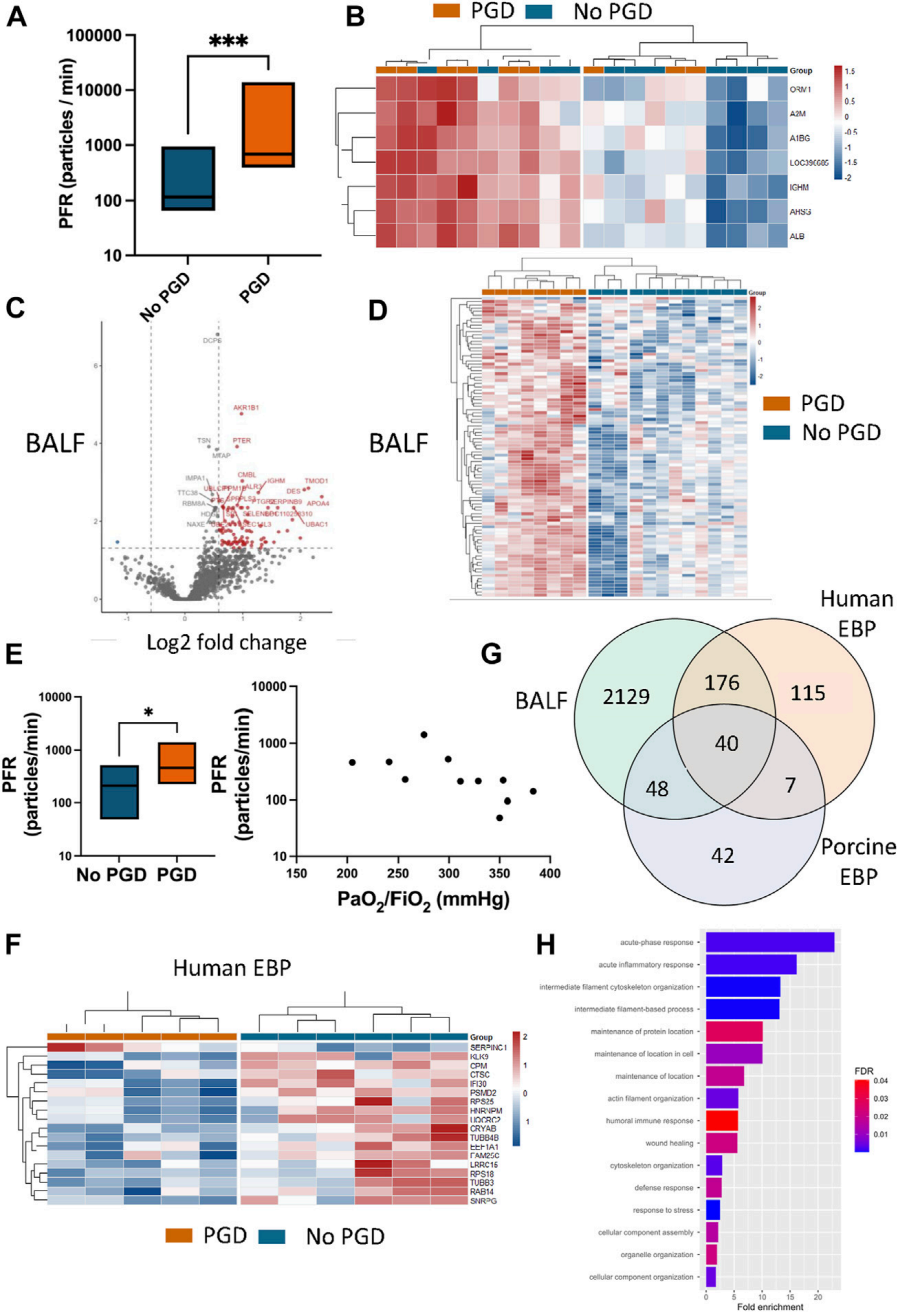
Particles in the exhaled breath of porcine and human transplant recipients with and without primary graft dysfunction (PGD) exhibited significant differences in both particle flow rate and protein identity and recapitulated identities found in the bronchoalveolar lavage fluid (BALF). **(A)** Particle flow rate (PFR) relative to the grade of PGD determined for the porcine lung transplant recipients. Plots represent measurements taken at the experimental endpoint for recipients with PGD (*n* = 9) or without PGD (*n* = 13). **(B)** Proteins from the exhaled breath particles (EBP, *n* = 20) were isolated and analyzed using mass spectrometry and a heat map was made of differentially expressed proteins when the PGD porcine recipient group was compared to the non-PGD group. **(C)** Proteins were also identified from within the bronchoalveolar lavage fluid (BALF) samples of the porcine transplant recipients with the volcano plot showing the differentially expressed proteins with significantly higher expressed proteins in red. **(D)** A heat map was generated from the differentially expressed BALF proteins showing the grouping of the PGD and non-PGD porcine recipients. **(E)** The PFR was also measured within human transplant recipients (*n* = 11) between groups (left) and per recipient in correlation to the recipient’s PaO_2_/FiO_2_ ratio. **(F)** Isolated proteins from the human exhaled breath particles (*n* = 11) were analyzed by mass spectrometry and a heat map was generated from the differentially expressed proteins. **(G)** A Venn diagram shows the overlap of protein identities isolated from the porcine bronchoalveolar lavage (BAL) fluid compared to the porcine EBPs and the human EBPs. **(H)** Gene Ontology (GO) term analysis was performed on the human EBPs to identify the biological processes from within the samples, with relevant terms highlighted in the plot demonstrating the fold enrichment and the false discovery rate (FDR)-corrected *p*-value in the bar color. An enlarged version can be found in the Supplementary Materials (Supplementary Figure S1). Plots represent measurements taken at the experimental endpoint for porcine recipients with PGD (*n* = 9) or without PGD (*n* = 13) and for human recipients with PGD (*n* = 5) and without PGD (*n* = 6). Statistically significant differences between groups were tested with the two-tailed Mann-Whitney test. *p < 0.05, ***p < 0.001. CPM, carboxypeptidase M; CRYAB, α-crystallin B chain; EEF1A1, elongation factor 1-α 1; HNRNPM, heterogeneous nuclear ribonucleoprotein M; IFI30, γ -interferon-inducible lysosomal thiol reductase; KLK9, kallikrein-9; LRRC15, leucine-rich repeat-containing protein 15; PSMD2, 26S proteasome non-ATPase regulatory subunit 2; RAB14, ras-related protein Rab-14; RPS18, 40S ribosomal protein S18; RPS25, 40S ribosomal protein S25; SERPINC1, antithrombin-III; SNRPG, small nuclear ribonucleoprotein G; TUBB3, tubulin β-3 chain; TUBB4B, tubulin β-4B chain; UQCRC2, cytochrome b-c1 complex subunit 2.

Using the custom PExA device connected to the expiratory limb of the ventilator, PFR was measured in the post-transplant recipients and found to be significantly higher in those with PGD with a rate of 686.4 (449.7–8,824.0) ppm. Those without PGD had a rate of 116.6 (79.7–307.4) ppm (*p* = 0.0003, Figure 3A). The EBPs were collected on a membrane from which the proteins were extracted. After filtering for those present in at least 60% of the samples per group, 137 proteins were analyzed, of which 7 were significantly overexpressed in PGD. Hierarchical clustering of the differentially expressed proteins showed a clear separation of the PGD group from the non-PGD group (Figure 3B). In BALF collected from all recipients, from which 2,418 proteins were identified after filtering for proteins in at least 65% of the samples. Of these, 91 proteins were overexpressed in PGD samples compared to 55 in non-PGD samples (Figure 3C). Again, hierarchical clustering differentiated between the two recipient groups (Figure 3D).

These results were additionally compared to findings collected from human EBPs from transplant recipients. As previously reported [5], the PFR in human recipients with PGD was higher than in non-PGD recipients. When comparing recipients, the PGD recipients had a significantly higher PFR (461.2 with IQR 284–1,177 ppm) compared to the non-PGD group (210.5 ppm with IQR 95–220.2, *p* = 0.0424; Figure 3E). When PFR was plotted against each recipient’s PaO_2_/FiO_2_ ratio, there was a significant correlation (Spearman r = −0.7364, *p* = 0.0128, Figure 3E). While this relationship between disease and PFR has been noted previously, the proteins within this exhaled breath have never been analyzed. Within the human samples, 338 proteins were found after filtering, of which 18 were significantly differentially expressed in PGD and hierarchical clustering showed separation of the PGD and non-PGD samples (Figure 3F). Proteins found in the EBPs of both the human and porcine samples overlapped with those identified in BALF. When comparing the porcine EBP proteins to BALF, there was an overlap of 88 protein identities, representing 64.2% of all EBP proteins (Figure 3G). When comparing human EBPs to BALF, there was an overlap of 216 proteins or 63.9% of human EBP proteins (Figure 3G). A PANTHER overrepresentation test was performed to examine the significantly enriched pathways and 16 terms were identified (Figure 3H). Within the PGD group, acute phase and acute inflammatory responses were highlighted, in addition to several terms related to cytoskeletal and filament organization.

To understand how these processes are important in the production of EBPs and to highlight the mechanism by which EBPs are increased in the PGD group, further analysis of both the tissue and BALF was performed to demonstrate the alterations in the alveolar-capillary barrier.

### Proteins in the Adherens and Tight Junctions Are Underexpressed in Tissue and Overexpressed in BALF

**FIGURE 4:**
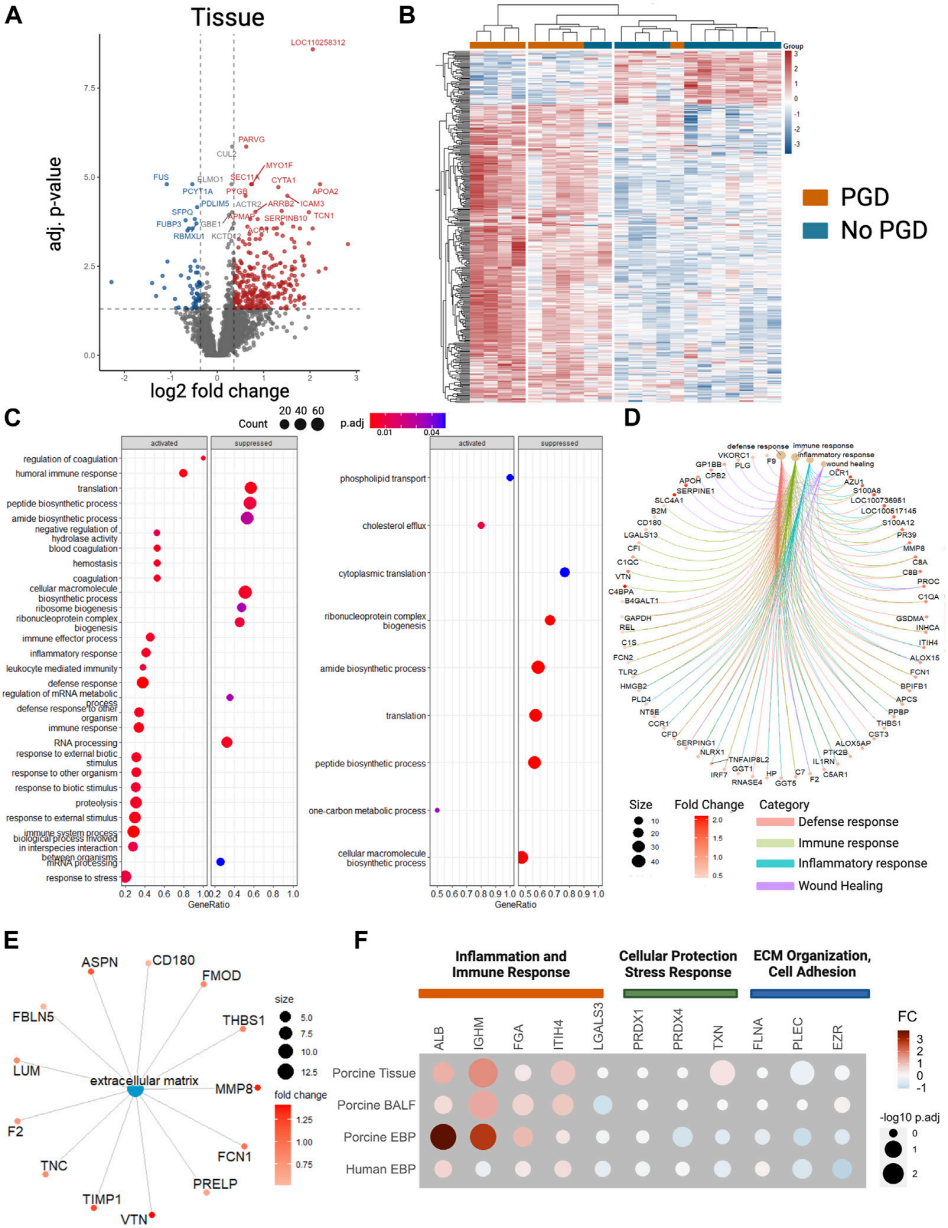
Proteins identified in the tissue between primary graft dysfunction (PGD) and no PGD samples showed differences in biological processes as identified by gene ontology analysis. **(A)** Volcano plots of differentially expressed proteins detected by mass spectrometry of porcine lung tissue samples at the experimental endpoint. Blue dots indicate proteins that were underexpressed in PGD samples while red dots are those that were overexpressed in the samples. **(B)** Heat maps of hierarchical clustering performed on porcine tissue samples on differentially expressed proteins with blue representing underexpression and red representing overexpression. **(C)** Gene set enrichment analysis (GSEA) was performed on the differentially expressed proteins to show the statistically significant biological processes found in the tissue (left) and BALF (right) samples represented as dot plots. An enlarged version can be found in the Supplementary Material (Supplementary Figure S2). **(D)** Gene-concept network plot showing the association between proteins and biological processes found in the GSEA. Refer to Supplementary Tables S1, S2 for a list of protein names. **(E)** Concept network plot for the analysis of the gene ontology (GO) term of the extracellular matrix. A list of protein names is in Supplementary Table S3. Enlarged versions of 4d and 4e can be found in the Supplementary Materials (Supplementary Figure S3). **(F)** The proteins identified were additionally compared between porcine tissue, BALF, and EBP samples and human EBP samples with a dot plot showing the fold change (FC) in color and the adjusted q-value in dot size. Proteins were grouped according to the GO term to which they belonged. Plots represent measurements taken at the experimental endpoint for recipients with PGD (*n* = 9) or without PGD (*n* = 13). Values represent the median and interquartile range (box) with minimum and maximum values (whiskers). Statistically significant differences between groups were tested with the two-tailed Mann-Whitney test. ***p < 0.001. ALB, albumin; EZR, ezrin; FGA, fibrinogen α chain; FLNA, filamin-A; IGHM, Immunoglobulin heavy constant mu; ITIH4, inter-α-trypsin inhibitor heavy chain H4; LGALS3, galectin; PLEC, plectin; PRDX1, peroxiredoxin-1; PRDX4, peroxiredoxin-4; TXN, thioredoxin.

Analysis of the lung tissue identified 5,206 proteins, of which 302 were significantly overexpressed in the PGD group and 55 were underexpressed (Figure 4A). As with other sample types like EBP and BALF, the hierarchical grouping of differentially expressed proteins showed a clear clustering of the PGD and non-PGD (Figure 4B). A GSEA was performed within the PGD group and showed that the enriched biological processes in the PGD samples included regulatory pathways of coagulation, wound healing, and responses to inflammation (Figure 4C). By isolating the specific pathways of defense responses, immune responses, inflammatory responses, and wound healing, enriched protein identities could be mapped to their biological processes (Figure 4D). Within the extracellular matrix, eleven proteins were significantly enriched, including known regulators such as metalloproteinase 8 (Figure 4E). Proteins identified in the tissue and BALF were additionally further compared to those found in the human and porcine EBPs (Figure 4F), demonstrating similarities in both the protein identities and relative fold changes. The identified proteins were grouped according to their corresponding GO biological processes.

**FIGURE 5:**
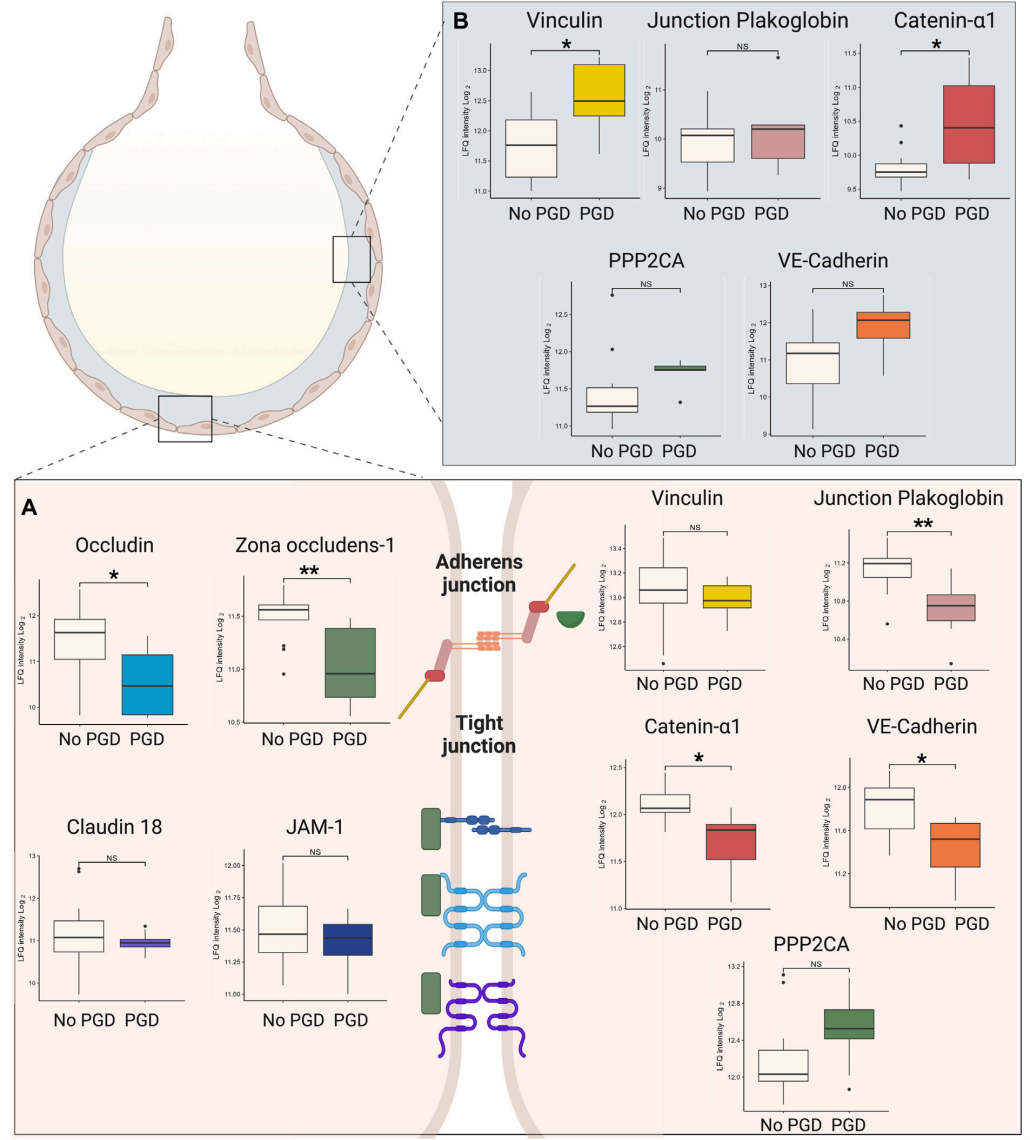
Differential expression of junctional proteins in the adherens junctions and the tight junctions in the tissue from porcine recipients with primary graft dysfunction (PGD). **(A)** Plots represent the differences found within individual analyses of protein expression in porcine tissue samples from no PGD and PGD samples with respect to tight junction (left) and adherens junction (right) proteins. **(B)** Plots of the differences found within individual analyses of protein expression in bronchoalveolar lavage fluid (BALF) from no PGD and PGD samples. Plots represent measurements taken at the experimental endpoint for recipients without PGD (*n* = 13) or with PGD (n = 9). Values represent the median and interquartile range (box) with minimum and maximum values (whiskers). Statistically significant differences are reported as FDR corrected *p*-values (q-values) using log (2)-fold change differences between groups (see Supplementary Methods), *q < 0.05, NS. JAM-1, junctional adhesion molecule-1; VE-cadherin, vascular endothelial cadherin; PPP2CA, serine/threonine-protein phosphatase 2A-catalytic subunit. Figure created in biorender.com.

To elucidate how there was a greater amount of exhaled breath particles in the respiratory tract lining fluid in the setting of PGD, the cell-cell adhesion proteins were examined. In the adherens junctions in the tissue samples (Figure 5A), junctional plakoglobin [log2 (FC) = −0.42, q = 0.005], catenin-α 1 [log2 (FC) = −0.36, q = 0.01], and vascular endothelial cadherin [log2 (FC) = −0.37, q = 0.02] were significantly underexpressed in the PGD group. Serine/threonine-protein phosphatase 2A catalytic subunit (PPP2CA) was higher in tissue from PGD samples, although not to a statistically significant degree. Zona occludens-1 and occludens of the tight junction were significantly underexpressed in PGD {log2 (FC) = −0.46, q = 0.005; [log2(FC) = −0.83, q = 0.04] respectively}, and others showed lower but not statistically significant levels, including junctional adhesions molecule-1 (JAM-1), claudin-18 and vinculin.

Within BALF (Figure 5B), adherens junction proteins were significantly higher in PGD samples. These included vinculin [log2 (FC) = 0.74, q = 0.04] and catenin-α 1 [log2 (FC) = 0.65, q = 0.03]. Junctional plakoglobin, PPP2CA, and vascular endothelial cadherin all showed increased but not statistically significant levels.

**FIGURE 6:**
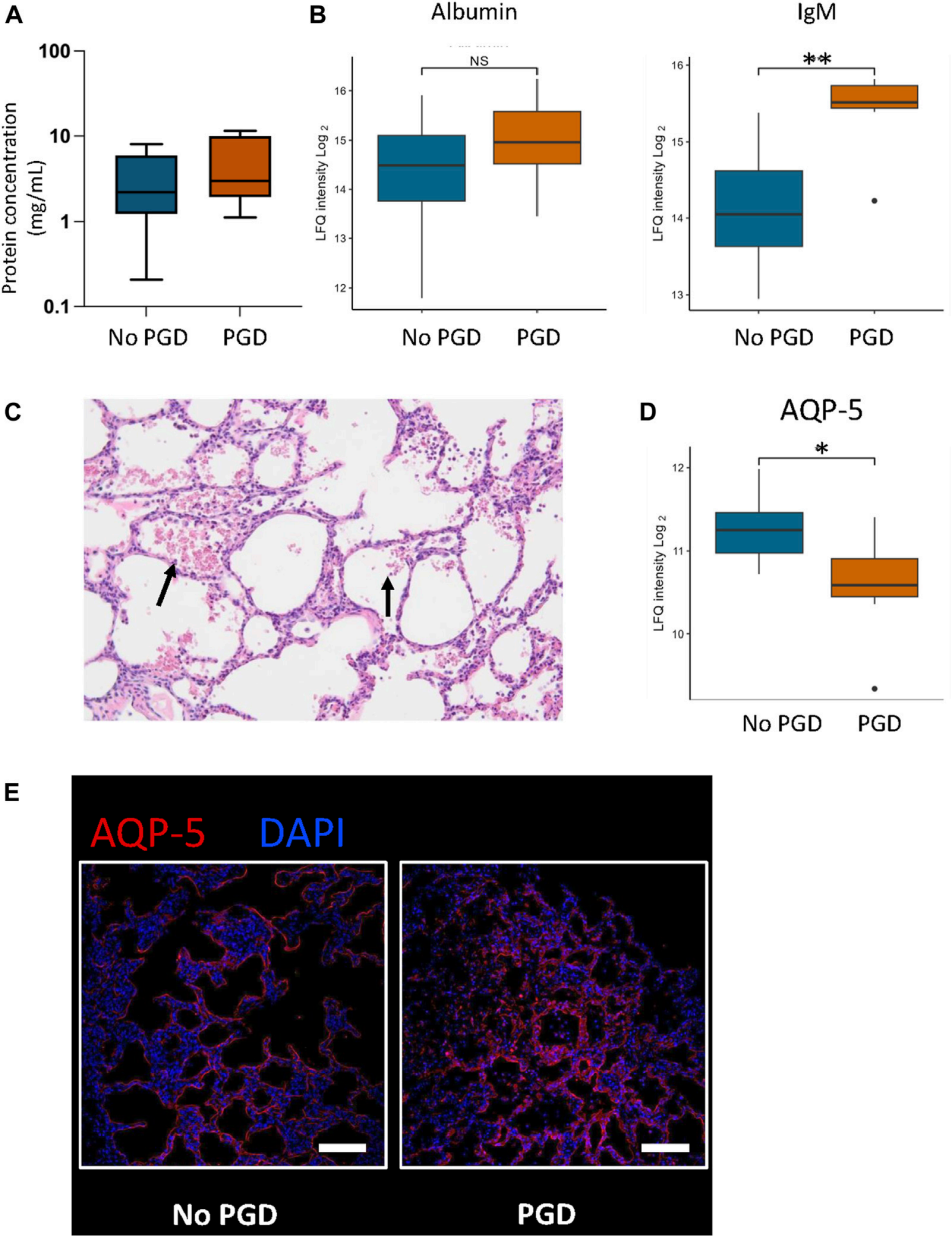
Changes in the alveolar-capillary barrier compared between porcine recipients with and without primary graft dysfunction (PGD). **(A)** Total protein concentration in bronchoalveolar lavage fluid (BALF) was measured in the recipients at the end of the experiment. **(B)** Expression levels of albumin and IgM were measured using mass spectrometry in BALF samples. **(C)** Representative image of red blood cells seen in the airspace (black arrows) of hematoxylin and eosin (H&E) stained lung tissue from a recipient with primary graft dysfunction. **(D)** Aquaporin-5 (AQP-5) was measured by mass spectrometry in the tissue (left) and then visualized by immunofluorescence staining. **(E)** shows representative images from a no PGD sample (left) and PGD sample (right) using immunofluorescence staining (4′,6-diamidino-2-phenylindole or DAPI in blue, AQP-5 in magenta). The scale bar represents 100 μm. Plots represent measurements taken at the experimental endpoint for recipients with PGD (*n* = 9) or without PGD (*n* = 13). Values represent the median and interquartile range (box) with minimum and maximum values (whiskers). Statistically significant differences are reported as FDR corrected *p*-values (q-values) using log (2)-fold change differences between groups (see Supplementary Methods)**q < 0.05, NS.

BALF was further examined to confirm the proteomic findings of alterations in the alveolar-capillary barrier. Total protein content in BALF showed an increasing trend toward the PGD group [2.0 (1.8–10.0) mg/mL in the PGD group, 2.2 (1.2–5.9) mg/mL in the non-PGD group, *p* = 0.1213, Figure 6A]. Albumin and IgM protein were overexpressed in the PGD BALF, demonstrating leakage of large molecular weight serum proteins, an established measure of alveolar-capillary barrier dysfunction [23] (Figure 6B). Other signs of alveolar-capillary barrier changes were found on histological examination, including H&E staining showing erythrocytes in the airspace in 6 of 9 PGD recipients (Figure 6C). Expression of aquaporin-5 (AQP-5), which is differentially localized to the apical membrane of the superficial epithelium in the airways, was decreased in tissue from the PGD group [log2 (FC) = −0.46, q = 0.04], which was also observed qualitatively by immunofluorescence imaging (Figures 6D, E).

## Discussion

Despite improvements in transplantation, PGD remains a threat to the postoperative recipient. Current methods of clinical appraisal could be supported by a non-invasive bedside approach to diagnostic surveillance. From this perspective, the addition of EBP analysis provides a novel means to monitor PGD, both from the rapidity of flow rate measurements that can be performed at the bedside and from the granular data that can be gathered from the in-depth analysis possible with collected particles. This study demonstrates that high EBP flow rates are significantly correlated with PGD and that the evaluation of proteins found in these EBPs can offer a window into the pathophysiological study of the distal airways without the need for more invasive bronchoscopy or tissue sampling. In this study, porcine and human EBP collections not only showed a correlation between high PFR and PGD incidence, but the proteins identified within the EBP samples reflected the BALF contents. Analysis of the tissue and BALF showed a disease-based difference in protein expression demonstrating alveolar-capillary barrier changes that explain the mechanism behind high PFR in PGD recipients.

Using the PExA device, breath particles are impacted according to their inertia, allowing both quantification and collection on a membrane housed within the device. In this study of a porcine lung transplant, PFR was correlated with the development of PGD at postoperative day 3. Previous studies have shown that PFR correlates with lung injury [8, 9] and a pilot study showed higher PFR in human lung transplant recipients with PGD [5]. In that report, we had previously reported that in human lung transplant recipients, particle counts measured on days 0, 1, 2, and 3 were increased in recipients with PGD compared to those without PGD [5]. In the present study, we aimed to build on these findings by identifying the proteins collected in exhaled breath and the mechanism by which PFR is higher in this state of injury, which has not been shown before. This work is novel not only for its proteomic profiling of the proteins captured by exhaled breath, but also because it aims to substantiate the hypothesis that changes to the alveolar-capillary barrier contribute to the PGD disease state and higher PFR through the analysis of both BALF and tissue samples. The evidence that porcine and human EBP particles recapitulate proteins found in BALF emphasizes that EBP collection can be a non-invasive means of sampling the distal airway without having to resort to bronchoalveolar lavage.

To understand why PFR was higher in the PGD group, an analysis of the alveolar-capillary barrier was pursued to demonstrate how injury status correlates with leakage of proteins into the respiratory tract lining fluid. While other forms of acute lung injury have demonstrated changes to the endothelial and epithelial barriers [23], this type of damage has not been as clearly established in studies of PGD, largely due to the lack of proteomic profiling in this disease. Other studies of the alveolar-capillary barrier have shown that tight and adherens junctions are important in maintaining alveolar permeability, with claudin-18 knockouts showing increased paracellular alveolar permeability [24, 25] and loss of vascular endothelial cadherin (VE-cadherin) implicated as a major mechanism of increased permeability in acute respiratory distress syndrome (ARDS) [26]. In this study, zona occludens, an important member of tight junctions, was significantly underexpressed in PGD tissue. Additionally, from the adherens junctions, VE-cadherin and its associated proteins including vinculin, junctional plakoglobin, catenin- α 1 and serine/threonine phosphatase 2 catalytic subunits were decreased in the PGD tissue in this study. This was accompanied by a concomitant increase in their BALF levels. The GO term “adherens junction assembly” was also statistically enriched within the EBP analysis.

Further evidence of alveolar-capillary barrier breakdown was found in the increased protein content of BALF and the presence of high molecular weight proteins, a recognized sign of barrier breakdown [23]. On H&E staining, erythrocytes were found in the airspace of the majority of PGD samples, further demonstrating barrier breakdown. AQP-5 levels were significantly reduced in the PGD group, which is important due to the specific localization of the protein to the apical surface of the lung epithelium, specifically within alveolar type 1 cell [27]. These results show that PGD status is correlated with significant changes in alveolar-capillary permeability. Combined with the findings of significant changes in tight and adherens junction components, the results that the alveolar-capillary barrier was significantly damaged in PGD can then be correlated to explain the finding of higher particle flow in the lining fluid of these recipients. This establishes the mechanism behind the higher PFR within this injury state.

There are few other proteomic studies of PGD in lung transplantation either in humans or in large animal models. Previous studies have primarily focused on the search for relevant biomarkers, which are typically measured in plasma or BALF. Individual proteins have been singled out instead [14, 28, 29], and thus this study demonstrates a novel use of proteomic profiling using mass spectrometry as a means to investigate the disease state through broader changes within the proteome. Mass spectrometry has rarely been utilized in lung transplantation research, with the exception of a few studies focusing on long-term outcomes in bronchiolitis obliterans syndrome [17, 30, 31]. This study therefore represents a novel approach to the study of PGD. Given the suggestion that there may be different phenotypes of PGD with different mechanisms underlying lung pathology, broader proteomic views such as those provided by EBP analysis may give a more detailed understanding of PGD pathophysiology [32]. Future endeavors with EBP collection and analysis could focus on expanding the findings within our porcine and human lung transplant recipients to look at proteomic changes in larger cohorts. This would be particularly valuable to increase the generalizability of the results as the current study only included human PGD grade 2 recipients. As implementation moves forward, EBP collection may become a complement to diagnostic techniques, but would need consideration and exercise of clinical judgment as an alternative to some techniques such as bronchoscopy, which, for example, may still be indicated for other reasons, such as mucus clearance and viral and bacterial sampling.

In conclusion, the use of exhaled breath particles allows for the rapid detection of PGD in lung transplant recipients by PFR measurement and may facilitate more in-depth analyses to investigate disease pathology by proteomic analysis of the distal airways. The higher PFR in this study in the PGD group coupled with the results showing the overlap between proteins captured by the EBPs compared to BALF sampling demonstrates that EBP collection can be an important diagnostic tool in the postoperative recipient. The advantages of such a technique include the ease with which the device can be connected to mechanical ventilation in addition to its lack of invasiveness, which is an improvement over traditional bronchoscopy. This technique can be implemented in clinical settings as a bedside diagnostic tool, thus allowing a transplant recipient to be monitored for the development of PGD in a convenient manner which can be leveraged for both rapid detection and more time-consuming but in-depth proteomic analysis.

## Data Availability

The datasets presented in this study can be found in online repositories. The names of the repository/repositories and accession number(s) can be found below: http://www.proteomexchange.org/, PXD046365.

## Footnotes

1
pantherdb.org

## Abbreviations

AQP-5, Aquaporin-5; BALF, Bronchoalveolar lavage fluid; BOS, Bronchiolitis obliterans syndrome; CLAD, Chronic lung allograft dysfunction; DIA, Data-independent acquisition; EBP, Exhaled breath particles; ECM, Extracellular matrix; GSEA, Gene set enrichment analysis; GO, Gene ontology; H&E, Hematoxylin and eosin; ICU, Intensive care unit; JAM-1, Junctional adhesion molecule-1; ISHLT, International Society for Heart and Lung Transplantation; LEA, Lycopersicon Esculentum lectin; LTx, Lung transplantation; MQ, Morphological quotient; PANTHER, Protein analysis through evolutionary relationships; PExA, Particles in exhaled air; PFR, Particle flow rate; PGD, Primary graft dysfunction; PPM, Particles per minute; PPP2CA, Serine/threonine-protein phosphatase 2A catalytic subunit; RTLF, Respiratory tract lining fluid.
