# Supplementary material for: Proteomic Analysis of Primary Graft Dysfunction in Porcine Lung Transplantation Reveals Alveolar-Capillary Barrier Changes Underlying the High Particle Flow Rate in Exhaled Breath
Source: Transpl Int. 2024 Apr 8;37:12298. doi: 10.3389/ti.2024.12298 (PMC11089893; doi:10.3389/ti.2024.12298)
Supplement: Supplementary file 1 [file DataSheet1.docx]

**Supplement**

*Supplementary methods*

*Animal Preparation*

Forty-four male and female adult farm-raised wild-type American Yorkshire pigs (*Sus scrofa domesticus*) were used in this study, a subset of which have been previously reported on with alternate variables and outcomes not re-reported in this publication. Blood type was determined using Seraclone™ Anti-A (blood grouping reagent, Bio-Rad, Medical Diagnostics GmbH, Dreieich, Germany) prior to the experiment. Donor and recipient pairs were matched according to blood type and weight. All were premedicated with xylazine (Rompun® vet. 20 mg/mL; Bayer AG, Leverkusen, Germany; 2 mg/kg) and ketamine (Ketaminol® vet. 100 mg/mL; Farmaceutici Gellini S.p.A., Aprilia, Italy; 20 mg/kg). A peripheral intravenous (IV) line was inserted in the earlobe, and a urinary catheter was inserted in the bladder. General anaesthesia was accomplished with ketamine (Ketaminol® vet), midazolam (Midazolam Panpharma®, Oslo, Norway) and fentanyl (Leptanal®, Lilly, France) infusions. Mechanical ventilation was established using a Siemens-Elema ventilator (Servo 900C, Siemens, Solna, Sweden). The animals were intubated with a 7.5 size endotracheal tube. The ventilator was set to volume-controlled ventilation (VCV) with the flow pattern switch in “constant flow” which lowers the peak pressures according to the manufacturer’s instructions. Inspiration time is set to 25% with pause time 10% to give an I:E ratio of 1:2. Ventilation was adjusted to maintain carbon dioxide levels (PaCO_2_) between 33 – 41 mmHg. Tidal volume (Vt) was kept at 6-8 mL/kg. Dynamic compliance was calculated by the equation $C_{dyn}= \frac{V_{T}}{(peak pressure-PEEP)}$. A pulmonary artery catheter (Swan-Ganz CCOmbo V and Introflex, Edwards Lifesciences Services GmbH, Unterschleissheim, Germany) was inserted in the right internal jugular vein and an arterial line (Secalon-T^TM^, Merit Medical Ireland Ltd, Galway, Ireland) was placed in the right common carotid artery. 21 pigs were used as donor pigs and 21 pigs were used as recipients. Dihydrostreptomycinsulfate (0.1 mL/kg) (Boehringer Ingelheim Animal Health Nordics A/S, Copenhagen, Denmark) was given subcutaneously before initiation of surgery in all animals.

*Arterial blood gases*

Arterial blood gases were analyzed every hour with an ABL 90 FLEX blood gas analyzer (Radiometer Medical ApS, Brønshøj, Denmark), and normalized to the blood temperature of 37°C according to clinical standards in the recipient animals.

*Hemodynamics*

Hemodynamic parameters were measured every hour in the recipient animals using thermodilution with a Swan-Ganz catheter and an arterial line. Heart rate (HR), systolic blood pressure (SBP), diastolic blood pressure (DBP), mean arterial pressure (MAP), central venous pressure (CVP), cardiac output (CO), systolic pulmonary pressure (SPP), diastolic pulmonary pressure (DPP), mean pulmonary pressure (MPP), pulmonary artery wedge pressure (PAWP), systemic vascular resistance (SVR), and pulmonary vascular resistance (PVR) were recorded.

*Pulmonary harvest in the donor*

A median sternotomy was performed and the pulmonary artery was cannulated via the right ventricle with a 28 F cannula secured by a purse string suture placed in the outflow tract of the pulmonary artery. A clamp was put on the superior vena cava, the inferior vena cava, and on the ascending aorta. The left atrium and inferior vena cava were opened. The lungs were perfused antegradely with 4 L of cold Perfadex® PLUS solution (XVIVO perfusion, Gothenburg, Sweden) distributed at a low perfusion pressure (< 20 mmHg). The lungs were harvested *en bloc* in a standard fashion. The lungs were immersed in cold Perfadex® solution.

*Left lung transplantation in the recipient*

The lung transplantation was performed according to the protocol described by Mariscal et al^1^. In brief, the pulmonary hilum was dissected through a left thoracotomy and the left pulmonary artery, left atrium, and left bronchus were clamped individually. A left pneumonectomy of the native left lung was performed thereafter. The donor lung was sewn in, and the anastomosis of the bronchus was sutured using polydioxanone sutures (PDS 4-0, Ethicon, Somerville, NJ, USA). The atrial cuff and the pulmonary artery were sutured with polypropylene (Prolene 5-0, Ethicon, Somerville, NJ, USA) using a continuous pattern. All animals were immunosuppressed using tacrolimus (0.15 mg/kg, orally) (Sandoz AS, Copenhagen, Denmark), and methylprednisolone sodium succinate (1 mg/kg, intravenously) (Solumedrol, Pfizer, New York, USA)*.* After suturing the bronchus, a bronchoscopy was done to confirm an open bronchial anastomosis.

*Recipient follow up*

The recipient animals were kept under anaesthesia using ketamine (Ketaminol® vet, Intervet AB, Stockholm, Sweden), midazolam (Midazolam Panpharma®, Panpharma Nordic, Oslo, Norway), fentanyl (Leptanal®, Piramal Critical Care B.V., Lilly, France), and rocuronium bromide (Esmeron®, Merck, Kenilworth, NJ, USA) infusions. 500 mg imipenem (Merck & Co. Inc., Kenilworth, NJ, USA) was given intravenously 3 times daily throughout the experimental timeframe. Dihydrostreptomycinsulfat (0.1 mL/kg) (Boehringer Ingelheim Animal Health Nordics A/S, Copenhagen, Denmark) was given subcutaneously once daily. All animals were continuously immunosuppressed using tacrolimus (0.15 mg/kg, PO) (Sandoz AS, Copenhagen, Denmark), and methylprednisolone (1 mg/kg, intravenously, twice daily) (Solumedrol, Pfizer, New York, USA)*.* The recipient ventilatory strategy included the use of lowest possible pressures while maintaining adequate oxygenation and ventilation. This included maintaining a PEEP between 5-10 cmH_2_O and a peak pressure below 30 cmH_2_O.

*Right pneumonectomy*

The pulmonary hilum was dissected through a mid-sternotomy, and a right pneumonectomy (including the accessory lobe) was performed to assess isolated function of the transplanted left lung. The recipient was followed for additional 4 hours using a Swan-Ganz catheter. While the recipient was under one lung ventilation, the tidal volume and respiratory rate were adjusted to maintain a peak pressure less than 30 cmH_2_O.

*Particles in Exhaled Air (PExA) and Exhaled Breath Particles (EBP)*

A customized PExA 2.0 device (PExA, Gothenburg, Sweden) was used in conjunction with mechanical ventilation and connected to the expiratory limb of the ventilator, as previously described^2^. The PExA device measured particle count as a particle flow rate (PFR, particles/minute) with the particles collected onto a membrane, measured as total accumulated mass (ng) of particles from the airways, as previously described^2^. Measured particles were in the range of 0.33-3.67 µm in diameter. Particles collected onto a membrane for biochemical analysis are referred to as exhaled breath particles (EBP). EBP samples were kept frozen at -80° C until analysis.

*Primary graft dysfunction staging*

The primary graft dysfunction (PGD) was staged according to the PaO_2_/FiO_2_ ratio using the ISHLT guidelines^3^. Presence of lung infiltrate to fulfill the criteria of PGD was assessed through imaging conducted with a mobile C-arm x-ray machine (Siemens, Munich, Germany).

*Histopathological analyses*

Biopsies were taken from the right lower lobe after intubation from the right lower lobe and from the transplanted left lung after completion of the experiment and fixed in 10% neutral buffered formalin solution (Sigma Aldrich, Merck KGaA, Darmstadt, Germany) at 4°C for forty-eight hours.

For histopathological staining, formalin-fixed tissue was processed in a graded ethanol series and isopropanol (both Fisher Scientific, UK) series and embedded in paraffin (Histolab, Askim, Sweden). 5 μm sections were cut, deparaffinized, and stained with hematoxylin and eosin (Merck Millipore, Darmstadt, Germany) and mounted with Pertex (Histolab, Sweden). Bright-field images were acquired with an Olympus CKX53 microscope (Olympus Life Sciences, Tokyo, Japan). Images from each recipient were assessed by two blinded scorers with experience in porcine lung injury. Features of alveolar wall thickening, hemorrhage, acute inflammation, chronic inflammation, capillary congestion, and alveolar macrophages were scored on a scale of 0 to 3 and the sums were calculated to report a lung injury score.

For immunofluorescent staining and analysis, 200 μm tissue sections were cut using a vibrating microtome (Leica VT1000S Vibrating Microtome, Leica Microsystems, Wetzlar, Germany). To provide structural support for sectioning, samples were embedded in 3% low melting point agarose in distilled water. Free floating sections were stained with DAPI (1:1000) and Lycopersicon Esculentum lectin (LEA), DyLight-488 (1:500), and aquaporin-5 (1:250) in PBS. Sections were incubated in staining solution for 30 minutes at room temperature on a rocking table and then washed 3 times in PBS. Sections were mounted on slides with fluoromount media. Imaging was carried out on a Nikon A1RHD confocal microscopy platform equipped with a Piezo stage and 20X objective (Nikon, Tokyo, Japan). To capture individual alveoli with distinct clarity a Nyquist-3 function was employed on acquisition. All images were captured at a resolution of 1024x1024 pixels, 208.39 nm/pixel. Nikon files were exported as 8-bit tiff images, one per channel, for analysis. Alveoli were individually imaged at random locations across sections from 5 animals. For baseline versus lung injury comparisons 10 alveoli per animal were imaged at random locations across the section.

*Image Analysis*

All immunofluorescent images were analyzed using Fiji software^4^. A polygon selection tool was used to trace both the outer and inner alveolar borders. To calculate wall thickness, as a function of total alveolar area, the area of the inner border was subtracted from the area of the outer border, and this value was expressed as a percent of the surface area of the outer border. The circularity metric was applied to the inner border using the *shape descriptors* measurement function. Morphological quotient (MQ) was calculated by dividing the circularity of a given alveolus by its wall thickness.

*Collection of EBPs in human lung transplantation patients*

Membranes with collected exhaled breath particles (EBPs) were included from 11 patients who underwent lung transplantation. All patients signed written informed consents and approval was given by the Ethics Committee for Research (Dnr 2017/396). Particles were collected using a modified PExA 2.0 instrument connected to mechanical ventilation while patients were in the intensive care unit post-transplant, as previously described^5^. This involved the connection of the device to the outflow tract of the respiratory circuit using a non-rebreathing valve from the tracheal tube to the inflow and outflow of the circuit. PGD was graded according to the ISHLT guidelines based on blood gas measures, ventilator settings and chest radiography.

*Mass spectrometry analysis*

*Sample preparation and protein digestion*

Protein was extracted from homogenized tissue, BALF, and the EBP membranes. Proteins were solubilized in 2% SDS and a BCA assay (Pierce, Thermo Fisher Scientific, Waltham, Massachusetts, USA) was performed to determine protein concentration for BALF and Tissue. 100 µg of protein was digested using an S-TRAP digestion protocol. Samples were reduced with 20 mM dithiothreitol (DTT) for 45 minutes at 56° C and then incubated with 40 mM iodoacetamide (IAA) in the dark at room temperature for 30 minutes. Samples were acidified with 2.5% phosphoric acid and washed with buffer before binding to an S-Trap CO_2_-micro-80 column (ProTifi, Fairport, New York, USA). Samples were double digested overnight at 37°C with lysine-C (Promega Mass spec grade at a 1:50 ratio of enzyme to protein by ng) and trypsin (Promega sequence grade, at a 1:50 ratio of enzyme to protein by ng) and a BCA assay (Pierce, Thermo Fisher Scientific, Waltham, Massachusetts, USA) was performed to determine the peptide concentrations.

*Peptide mixing and pre-fractionation*

Fractionation was carried out using a Waters XBridge BH130 C18 3.5 μm, 2.1 × 150 mm column on an Ultimate 3000 RS HPLC (Thermo Scientific, Waltham, Massachusetts, USA) operating at 200 µL/min. The mobile phases were solvent A: 10 mM ammonium formate pH 10, and solvent B: 90% ACN and 10% water containing 10 mM ammonium formate pH 10. Peptides are separated using the following gradient: 0 min 0% B; 3 min 0% B, 97 min 35% B; 98 min 80% B; 108 min 80% B. The column was operated at RT and the detection wavelength was 214 nm. We collected 96 fractions at 1 min intervals which were further concatenated to 48 fractions by combining 2 fractions that are 24 fractions apart, i.e. #1 and #25; #2 and #26; etc. The fractions were dried in a Speed-Vac.

*LC-MS/MS data acquisition*

*DDA data acquisition on timsTOF Pro 2*

Fractions were resuspended in 0.1% formic acid and peptide determination was performed in a Nanodrop system (DeNovix, Wilmington Dellaware USA) before LC-MS/MS analysis. 400ng of each fraction were loaded on Evosep tips (Evosep Biosystems, Odense, Denmark) for separation with nanoflow reversed-phase chromatography with an EVOSEP ONE liquid chromatography (LC) system (Evosep Biosystems). Separation was performed with the 30 SPD method (gradient length 21 min) using a 15 cm x 150 µm Evosep column (Evosep Biosystems) packed with 1.5 μm ReproSil-Pur C18-AQ particles. The Evosep One was coupled to a timsTOF Pro 2 ion mobility mass spectrometer (Bruker, Billerica, MA, USA) operated in DDA PASEF with 10 PASEF scans per acquisition cycle and accumulation and ramp times of 100 ms each. Singly charged precursors were excluded, the ‘target value’ was set to 20,000 and dynamic exclusion was activated and set to 0.4 min. The quadrupole isolation width was set to 2 Th for m/z < 700 and 3 Th for m/z > 800. All subsequent DDA files were used to build a spectral library in Fragpipe v 18.0 ^6-9^ with the following parameters: Missed cleavages=2, Min peptide length=7, Max peptide length=50, and common internal retention time peptides (CiRT) were used for spectral library retention time calibration. Uniprot UP000008227 FASTA (release 2023_01) was used as a database with reversed target sequences as decoys. The generated library consisted of 10296 protein groups in total. A py_diAID method ^10^ was generated by subjecting the 48 DDA fraction runs, this method file was used to run all individual samples in the study in diaPASEF on the timsTOF Pro 2.

*DIA data acquisition on timsTOF Pro 2*

BALF and Tissue samples were loaded onto Evosep tips (Evosep Biosystems) and separated with the same gradient as for DDA data acquisition. MS data were acquired using the diaPASEF method. The accumulation and ramp times were set to 100 ms. DIA scans were acquired with 25 m/z isolation windows spanning 247-1350 m/z and 0.60-1.60 1/K0 ion mobility ranges and an estimated cycle time of 2.76s. The collision energy was ramped linearly as a function of the mobility from 59 eV at 1/K0 = 1.6 Vs cm-2 to 20 eV at 1/K0 = 0.6 Vs cm-2.

*DIA data acquisition on timsTOF SCP*

Protein digests were resuspended in 20 μL of 2% ACN/0.1% TFA containing iRT peptide standard and loaded onto Evotip Pure (Evosep Biosystems) according to the manufacturer´s instructions. The Evosep One liquid chromatography system coupled with a timsTOF SCP mass spectrometer via a CaptiveSpray nano-electrospray ion source (Bruker, Billerica, MA) was used to measure the samples. Separation was performed with the Whisper 40 SPD (samples per day) method using an Aurora Elite nanoflow UHPLC column with CSI fitting (15 cm x 75 μm ID, 1.7 μm C18) (IonOpticks, Fitzroy, VIC) at 50°C. The mobile phases comprised 0.1% FA as solution A and 0.1% FA in ACN as solution B. MS data was acquired using a diaPASEF method. The accumulation and ramp times were set to 100 ms. DIA scans were acquired with 25 m/z isolation windows spanning 400–1,000 m/z and 0.64–1.37 1/K0 ion mobility ranges. The method with 8 diaPASEF scans had a cycle time of 0.94 s. The collision energy was ramped linearly as a function of the mobility from 59 eV at 1/K0 = 1.6 Vs cm−2 to 20 eV at 1/K0 = 0.6 Vs cm−2. For low sample amounts high sensitivity detection was enabled.

*Bioinformatic analysis of LC-MS/MS data*

All diaPASEF runs were analyzed using DIA-NN v 1.8.1. Quantification mode was set to Robust LC (High Precision), with default RT-dependent normalization. For tissue and BALF the Fragpipe library was used while EBPs were searched library free with a concatenated FASTA file of UP000008227 (release 2023_01, uniprot.org) and human contaminants previously found on blank membranes. For the library free search, peptide lengths of 7 – 30 AA were considered, and precursor charge states in the range 2 – 4. FDR was set at 1%. Subsequently the output files were loaded into RStudio v 2022.12.0 with R v 4.2.2. For tissue and BALF the MS-DAP package^11^ was used for normalization and differential expression analysis using the following parameters: minimum detected peptides: 3, filtered at 65% identified proteins in all samples. Normalization was performed using variance stabilizing normalization for BALF and Tissue Differential expression was performed using the MSqRob R package^12^. Log2 foldchange thresholds were inferred through bootstrapping in the MS-DAP package. Significantly changed proteins were defined as FDR corrected p-values (q-values) > 0.05 and Log 2 fold change +/- 0.359 and 0.585 for tissue and BALF respectively. Due to the increased amounts of missing values in EBP membranes, an imputation strategy had to be employed and as such all human and porcine EBP membranes data were analyzed using the DEP R package (v 1.20.0)^13^. First human contaminants and proteins identified in less than 60% of samples in at least one group were filtered out. Next the MaxLFQ intensities were normalized using quantile normalization within the NormalyzerDE package (v 1.16.0)^14^. Next missing values where imputed based on a mixed imputation strategy with proteins not missing at random (MNAR) not identified in any samples of one group imputed using MinDet and proteins missing at random (MAR) imputed using maximum likelihood estimates (MLE). Differential expression analysis was run within the DEP package with alpha set to 0.05 and log 2 fold change +/- 0.7. For the heatmaps, MaxLFQ values were normalized using z-scores and plotted using the pheatmap package v 1.0.12 with euclidean clustering. GSEA analysis for Tissue and BALF protein data was performed using the clusterProfiler package v 4.4.4. For EBP a GO term enrichment analysis was performed using geneontology.orgs PANTHER GO Enrichment Analysis on all identified proteins used in the differential expression analysis.

*Calculations and statistics*

Continuous variables were reported as median and interquartile range (IQR). Statistically significant differences between groups were tested with the Student’s T-test when comparing two groups and within groups with ANOVA when data were normally distributed. Most analyses were conducted with the Mann-Whitney test and the Kruskal-Wallis tests as data were not normally distributed. A Chi-Squared test was performed to analyze observed frequencies of categorical variables. These statistical analyses were performed using GraphPad Prism 9.1. The statistics used within mass spectrometry analysis are reported in the “Bioinformatic analysis of LC-MS/MS data” section. Significance was defined as: p < 0.001 (***), p < 0.01 (**), p < 0.05 (*), and p > 0.05 (not significant).

**References**

1. Mariscal A, Caldarone L, Tikkanen J, et al. Pig lung transplant survival model. *Nat Protoc*. Aug 2018;13(8):1814-1828. doi:10.1038/s41596-018-0019-4

2. Broberg E, Andreasson J, Fakhro M, et al. Mechanically ventilated patients exhibit decreased particle flow in exhaled breath as compared to normal breathing patients. *ERJ Open Res*. Jan 2020;6(1)doi:10.1183/23120541.00198-2019

3. Snell GI, Yusen RD, Weill D, et al. Report of the ISHLT Working Group on Primary Lung Graft Dysfunction, part I: Definition and grading-A 2016 Consensus Group statement of the International Society for Heart and Lung Transplantation. *J Heart Lung Transplant*. Oct 2017;36(10):1097-1103. doi:10.1016/j.healun.2017.07.021

4. Schindelin J, Arganda-Carreras I, Frise E, et al. Fiji: an open-source platform for biological-image analysis. *Nat Methods*. Jun 28 2012;9(7):676-82. doi:10.1038/nmeth.2019

5. Broberg E, Hyllen S, Algotsson L, Wagner DE, Lindstedt S. Particle Flow Profiles From the Airways Measured by PExA Differ in Lung Transplant Recipients Who Develop Primary Graft Dysfunction. *Exp Clin Transplant*. Dec 2019;17(6):803-812. doi:10.6002/ect.2019.0187

6. da Veiga Leprevost F, Haynes SE, Avtonomov DM, et al. Philosopher: a versatile toolkit for shotgun proteomics data analysis. *Nat Methods*. Sep 2020;17(9):869-870. doi:10.1038/s41592-020-0912-y

7. Nesvizhskii AI, Keller A, Kolker E, Aebersold R. A statistical model for identifying proteins by tandem mass spectrometry. *Anal Chem*. Sep 1 2003;75(17):4646-58. doi:10.1021/ac0341261

8. Kong AT, Leprevost FV, Avtonomov DM, Mellacheruvu D, Nesvizhskii AI. MSFragger: ultrafast and comprehensive peptide identification in mass spectrometry-based proteomics. *Nat Methods*. May 2017;14(5):513-520. doi:10.1038/nmeth.4256

9. Kall L, Canterbury JD, Weston J, Noble WS, MacCoss MJ. Semi-supervised learning for peptide identification from shotgun proteomics datasets. *Nat Methods*. Nov 2007;4(11):923-5. doi:10.1038/nmeth1113

10. Skowronek P, Thielert M, Voytik E, et al. Rapid and In-Depth Coverage of the (Phospho-)Proteome With Deep Libraries and Optimal Window Design for dia-PASEF. *Mol Cell Proteomics*. Sep 2022;21(9):100279. doi:10.1016/j.mcpro.2022.100279

11. Koopmans F, Li KW, Klaassen RV, Smit AB. MS-DAP Platform for Downstream Data Analysis of Label-Free Proteomics Uncovers Optimal Workflows in Benchmark Data Sets and Increased Sensitivity in Analysis of Alzheimer's Biomarker Data. *J Proteome Res*. Feb 3 2023;22(2):374-386. doi:10.1021/acs.jproteome.2c00513

12. Sticker A, Goeminne L, Martens L, Clement L. Robust Summarization and Inference in Proteome-wide Label-free Quantification. *Mol Cell Proteomics*. Jul 2020;19(7):1209-1219. doi:10.1074/mcp.RA119.001624

13. Zhang X, Smits AH, van Tilburg GB, Ovaa H, Huber W, Vermeulen M. Proteome-wide identification of ubiquitin interactions using UbIA-MS. *Nat Protoc*. Mar 2018;13(3):530-550. doi:10.1038/nprot.2017.147

14. Willforss J, Chawade A, Levander F. NormalyzerDE: Online Tool for Improved Normalization of Omics Expression Data and High-Sensitivity Differential Expression Analysis. *J Proteome Res*. Feb 1 2019;18(2):732-740. doi:10.1021/acs.jproteome.8b00523

**Supplementary Figure 1.** Enlarged version of Figure 3h

**Supplementary**
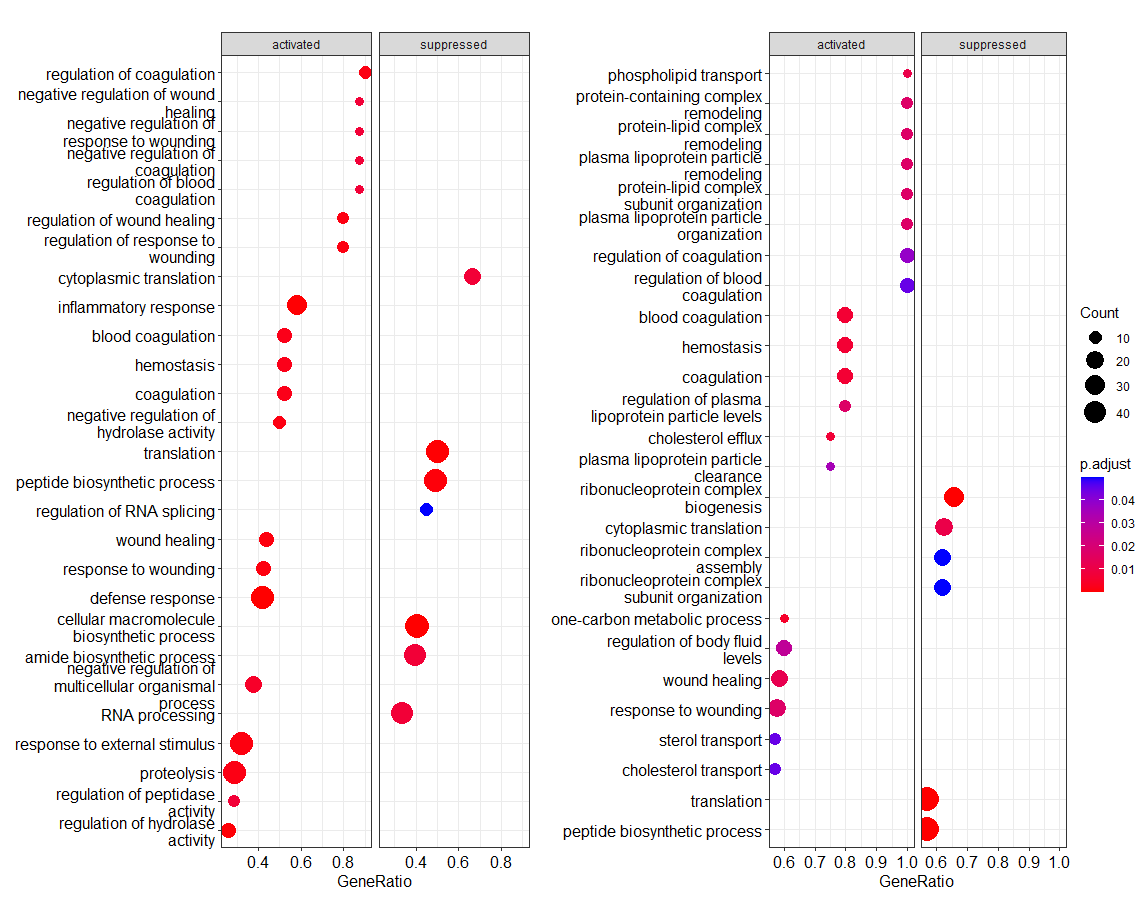
**Figure 2.** Enlarged version of Figure 4c


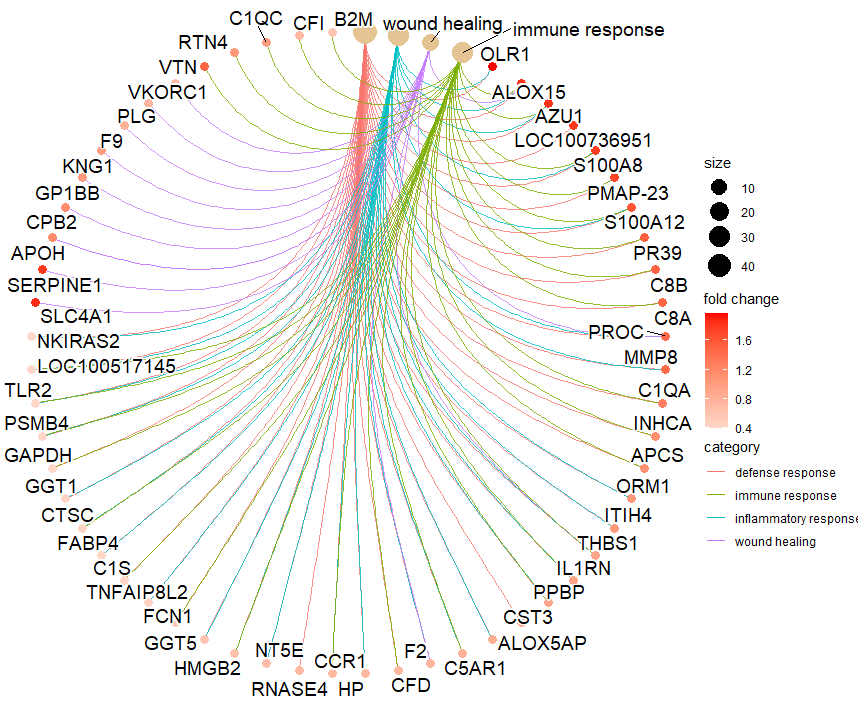


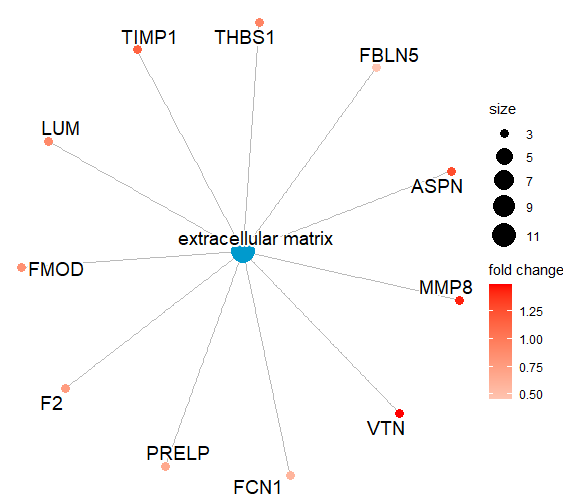


**Supplementary Figure 3.** Enlarged version of Figure 4d and 4e.

**Supplementary Table 1.** List of proteins examined in the concept network in Fig. 4d.

| Defense response | Immune response | Inflammatory response | Wound healing |  |
| --- | --- | --- | --- | --- |
| OLR1 | C4BPA | OLR1 | SLC4A1 | |
| AZU1 | AZU1 | AZU1 | SERPINE1 | |
| S100A8 | S100A8 | S100A8 | PROC | |
| LOC100736951 | LOC100517145 | LOC100517145 | APOH | |
| LOC100517145 | S100A12 | S100A12 | CPB2 | |
| S100A12 | PR39 | MMP8 | ALOX15 | |
| PR39 | VTN | PROC | GP1BB | |
| MMP8 | C8A | ITIH4 | THBS1 | |
| C8A | C8B | ALOX15 | F2 | |
| C8B | C1QA | PPBP | PLG | |
| PROC | INHCA | THBS1 | SERPING1 | |
| C1QA | ALOX15 | ALOX5AP | VKORC1 | |
| GSDMA | C1QC | IL1RN | F9 | |
| INHCA | FCN1 | C5AR1 |  |  |
| ITIH4 | BPIFB1 | F2 |  |  |
| ALOX15 | APCS | GGT5 |  |  |
| FCN1 | PPBP | HP |  |  |
| BPIFB1 | THBS1 | GGT1 |  |  |
| APCS | PTK2B | TNFAIP8L2 |  |  |
| PPBP | IL1RN | NLRX1 |  |  |
| THBS1 | C5AR1 | CCR1 |  |  |
| CST3 | C7 | NT5E |  |  |
| ALOX5AP | IRF7 | PLD4 |  |  |
| PTK2B | CFI | HMGB2 |  |  |
| IL1RN | NLRX1 |  |  |  |
| C5AR1 | SERPING1 |  |  |  |
| F2 | CFD |  |  |  |
| C7 | LGALS13 |  |  |  |
| GGT5 | CCR1 |  |  |  |
| HP | HMGB2 |  |  |  |
| RNASE4 | CD180 |  |  |  |
| GGT1 | TLR2 |  |  |  |
| IRF7 | FCN2 |  |  |  |
| TNFAIP8L2 | C1S |  |  |  |
| NLRX1 | B2M |  |  |  |
| SERPING1 | REL |  |  |  |
| CFD | GAPDH |  |  |  |
| CCR1 |  |  |  |  |
| NT5E |  |  |  |  |
| PLD4 |  |  |  |  |
| HMGB2 |  |  |  |  |
| TLR2 |  |  |  |  |
| FCN2 |  |  |  |  |
| C1S |  |  |  |  |
| REL |  |  |  |  |
| GAPDH |  |  |  |  |
| B4GALT1 |  |  |  |  |

**Supplementary Table 2.** List of protein abbreviations and corresponding names from the concept network in Fig. 4d.

| Protein Abbreviation | Uniprot Entry | Protein Name |
| --- | --- | --- |
| OLR1 | Q9TTK7 | Oxidized low-density lipoprotein receptor 1 |
| AZU1 | P80015 | Azurocidin |
| S100A8 | C3S7K5 | Protein S100 |
| LOC100736951 | F1SNU4 | Uncharacterized protein |
| LOC100517145 | A0A287A5Z8 | Complement C3 |
| S100A12 | P80310 | Protein S100-A12 |
| PR39 | P80054 | Antibacterial protein PR-39 |
| MMP8 | F1SV69 | Matrix metallopeptidase 8 |
| C8A | F1S788 | Complement C8 alpha chain |
| C8B | A0A287AT36 | Complement C8 beta chain |
| PROC | Q9GLP2 | Vitamin K-dependent protein C |
| C1QA | Q69DL0 | Complement C1q subcomponent subunit A |
| GSDMA | F1RXA6 | Gasdermin A |
| INHCA | I3LBF1 | Inhibitor of carbonic anhydrase |
| ITIH4 | P79263 | Inter-alpha-trypsin inhibitor heavy chain H4 |
| ALOX15 | P16469 | Polyunsaturated fatty acid lipoxygenase ALOX15 |
| FCN1 | Q29042 | Ficolin-1 |
| BPIFB1 | A0A5G2QRW6 | BPI fold containing family B member 1 |
| APCS | O19063 | Serum amyloid P-component |
| PPBP | P43030 | Platelet basic protein |
| THBS1 | F1SS26 | Thrombospondin 1 |
| CST3 | Q0Z8R0 | Cystatin C |
| ALOX5AP | P30356 | Arachidonate 5-lipoxygenase-activating protein |
| PTK2B | F1RJS6 | non-specific protein-tyrosine kinase |
| IL1RN | Q29056 | Interleukin-1 receptor antagonist protein |
| C5AR1 | I3LUE7 | C5a anaphylatoxin chemotactic receptor 1 |
| F2 | F1SIB1 | Prothrombin |
| C7 | Q9TUQ3 | Complement component C7 |
| GGT5 | F1RLR8 | Gamma-glutamyltransferase 5 |
| HP | Q8SPS7 | Haptoglobin [Cleaved into: Haptoglobin alpha chain; Haptoglobin beta chain] |
| RNASE4 | P15468 | Ribonuclease 4 |
| GGT1 | P20735 | Glutathione hydrolase 1 proenzyme |
| IRF7 | A0ZVR0 | Interferon regulatory factor 7 |
| TNFAIP8L2 | F1SSY0 | TNF alpha induced protein 8 like 2 |
| NLRX1 | F1SAH1 | NLR family member X1 |
| SERPING1 | F1SJW8 | Plasma protease C1 inhibitor |
| CFD | P51779 | Complement factor D |
| CCR1 | Q6YST0 | C-C motif chemokine receptor 1 |
| NT5E | K7GSR6 | 5'-nucleotidase |
| PLD4 | F1S9V1 | Phospholipase D family member 4 |
| HMGB2 | F1RJ01 | High mobility group box 2 |
| TLR2 | Q59HI8 | Toll-like receptor 2 |
| FCN2 | Q29041 | Ficolin-2 |
| C1S | A0A8W4FL59 | Complement C1s |
| REL | A0A287BI09 | REL proto-onco, NF-kB subunit |
| GAPDH | P00355 | Glyceraldehyde-3-phosphate dehydrogenase |
| B4GALT1 | A0A287BG16 | Beta-1,4-galactosyltransferase |
| C4BPA | A0A287A3T0 | Sushi domain-containing protein |
| VTN | P48819 | Vitronectin |
| C1QC | A0A286ZSJ7 | Complement C1q C chain |
| CFI | A0A287AQ20 | Complement factor I |
| LGALS13 | B7U2G5 | Galectin |
| CD180 | Q7YRL4 | CD180 antigen |
| B2M | Q07717 | Beta-2-microglobulin |
| SLC4A1 | K7GR72 | Anion exchange protein |
| SERPINE1 | P79335 | Plasminogen activator inhibitor 1 |
| APOH | A0A286ZFW3 | Beta-2-glycoprotein 1 |
| CPB2 | F1RK01 | Carboxypeptidase B2 |
| GP1BB | B7TY21 | Glycoprotein Ib platelet subunit beta |
| PLG | P06867 | Plasminogen |
| VKORC1 | A0A287B0I6 | vitamin-K-epoxide reductase |
| F9 | P16293 | Coagulation factor IX |

**Supplementary Table 3.** List of proteins with abbreviated names from the concept network in Fig 4e.

| Protein Abbreviation | Uniprot Entry | Protein Name |
| --- | --- | --- |
| CD180 | Q7YRL4 | CD180 antigen |
| FMOD | Q9TTB4 | Fibromodulin |
| THBS1 | F1SS26 | Thrombospondin 1 |
| MMP8 | F1SV69 | Matrix metallopeptidase 8 |
| FCN1 | Q29042 | Ficolin-1 |
| PRELP | F1S6B4 | Prolargin |
| VTN | P48819 | Vitronectin |
| TIMP1 | P35624 | Metalloproteinase inhibitor 1 |
| TNC | Q29116 | Tenascin |
| F2 | F1SIB1 | Prothrombin |
| LUM | F1SQ09 | Lumican |
| FBLN5 | F1SD87 | Fibulin 5 |
| ASPN | F1SUE4 | Asporin |
